# Community consensus on core open science practices to monitor in biomedicine

**DOI:** 10.1371/journal.pbio.3001949

**Published:** 2023-01-24

**Authors:** Kelly D. Cobey, Stefanie Haustein, Jamie Brehaut, Ulrich Dirnagl, Delwen L. Franzen, Lars G. Hemkens, Justin Presseau, Nico Riedel, Daniel Strech, Juan Pablo Alperin, Rodrigo Costas, Emily S. Sena, Thed van Leeuwen, Clare L. Ardern, Isabel O. L. Bacellar, Nancy Camack, Marcos Britto Correa, Roberto Buccione, Maximiliano Sergio Cenci, Dean A. Fergusson, Cassandra Gould van Praag, Michael M. Hoffman, Renata Moraes Bielemann, Ugo Moschini, Mauro Paschetta, Valentina Pasquale, Valeria E. Rac, Dylan Roskams-Edris, Hermann M. Schatzl, Jo Anne Stratton, David Moher

**Affiliations:** 1 University of Ottawa Heart Institute, Ottawa, Ontario, Canada; 2 School of Epidemiology and Public Health, Faculty of Medicine, University of Ottawa, Ottawa, Ontario, Canada; 3 School of Information Studies, Faculty of Arts, University of Ottawa, Ottawa, Ontario, Canada; 4 Scholarly Communications Lab, Ottawa and Vancouver, Canada; 5 Clinical Epidemiology Program, Ottawa Hospital Research Institute, Ottawa, Ontario, Canada; 6 Department of Experimental Neurology, Charité-Universitätsmedizin Berlin, Berlin, Germany; 7 QUEST Center for Responsible Research, Berlin Institute of Health at Charité-Universitätsmedizin Berlin, Berlin, Germany; 8 Department of Clinical Research, University of Basel and University Hospital Basel, Basel, Switzerland; 9 Meta-Research Innovation Center at Stanford (METRICS), Stanford University, Stanford, California, United States of America; 10 School of Psychology, Faculty of Social Science, University of Ottawa, Ottawa, Ontario, Canada; 11 Meta-Research Innovation Center Berlin (METRIC-B), Berlin Institute of Health, Berlin, Germany; 12 School of Publishing, Simon Fraser University, Vancouver, British Columbia, Canada; 13 Centre for Science and Technology Studies (CWTS), Leiden University, Leiden, the Netherlands; 14 Centre for Clinical Brain Sciences, University of Edinburgh, Edinburgh, United Kingdom; 15 Sport and Exercise Medicine Research Centre, La Trobe University, Melbourne, Victoria, Australia; 16 Department of Family Practice, University of British Columbia, Vancouver, British Columbia, Canada; 17 Douglas Research Centre, Montreal, Quebec, Canada; 18 Graduate Program in Dentistry, Federal University of Pelotas, Pelotas, Brazil; 19 Università Vita-Salute San Raffaele, Milano, Italy; 20 Wellcome Centre for Integrative Neuroimaging, University of Oxford, Oxford, United Kingdom; 21 Princess Margaret Cancer Centre, University Health Network, Toronto, Ontario, Canada; 22 Department of Medical Biophysics, University of Toronto, Toronto, Ontario, Canada; 23 Department of Computer Science, University of Toronto, Toronto, Ontario, Canada; 24 Vector Institute for Artificial Intelligence, Toronto, Ontario, Canada; 25 Graduate Program in Nutrition and Foods, Federal University of Pelotas, Pelotas, Brazil; 26 Istituto Italiano di Tecnologia, Genoa, Italy; 27 Department of Oncology, University of Turin, Turin, Italy; 28 Program for Health System and Technology Evaluation, Ted Rogers Centre for Heart Research at Peter Munk Cardiac Centre, Toronto General Hospital Research Institute (TGHRI), University Health Network (UHN), Toronto, Ontario, Canada; 29 Institute of Health Policy, Management and Evaluation (IHPME), Dalla Lana School of Public Health, University of Toronto, Toronto, Ontario, Canada; 30 Diabetes Action Canada, CIHR SPOR Network, Toronto, Canada; 31 Montreal Neurological Institute, McGill University, Montreal, Quebec, Canada; 32 Tanenbaum Open Science Institute, McGill University, Montreal, Quebec, Canada; 33 Department of Comparative Biology & Experimental Medicine, University of Calgary, Calgary, Alberta, Canada

## Abstract

The state of open science needs to be monitored to track changes over time and identify areas to create interventions to drive improvements. In order to monitor open science practices, they first need to be well defined and operationalized. To reach consensus on what open science practices to monitor at biomedical research institutions, we conducted a modified 3-round Delphi study. Participants were research administrators, researchers, specialists in dedicated open science roles, and librarians. In rounds 1 and 2, participants completed an online survey evaluating a set of potential open science practices, and for round 3, we hosted two half-day virtual meetings to discuss and vote on items that had not reached consensus. Ultimately, participants reached consensus on 19 open science practices. This core set of open science practices will form the foundation for institutional dashboards and may also be of value for the development of policy, education, and interventions.

## Abstract 2

The state of open science needs to be monitored to track changes over time and identify areas to create interventions to drive improvements. This Consensus View identifies 19 open science practices that will form the foundation for institutional dashboards for monitoring progress.

## Introduction

In November 2021, UNESCO adopted its Recommendation on Open Science, defining open science “as an inclusive construct that combines various movements and practices aiming to make multilingual scientific knowledge openly available, accessible and reusable for everyone, to increase scientific collaborations and sharing of information for the benefits of science and society, and to open the processes of scientific knowledge creation, evaluation and communication to societal actors beyond the traditional scientific community” [1]. UNESCO recommends that its 193 member states take action towards achieving open science globally. The recommendation emphasizes the importance of monitoring policies and practices in achieving this goal [1]. Open science provides a means to improve the quality and reproducibility of research [2,3], and a mechanism to foster innovation and discovery [4,5]. The UNESCO Recommendation has cemented open science’s position as a global science policy priority. It follows other initiatives from major research funders, such as the Open Research Funders Group, as well as national efforts to implement open science via federal open science plans [6,7].

Despite these commitments from policymakers and funders, adopting and implementing open science has not been straightforward. There remains debate about how to motivate and incentivize individual researchers to adopt open science practices [8–10], and how to best track open science practices within the community. A key concern is the need for funding to cover the additional fees and time costs needed to adhere to some open science best practices, when the academic reward system and career advancement still incentivize traditional, closed research practices. What “counts” in the tenure process is typically the outwardly observable number of publications in prestigious—typically high impact factor and often paywalled—journals, rather than efforts towards making research more accessible, shareable, transparent, and reusable. Monitoring open science practices is essential if the research community intends to evaluate the impact of policies and other interventions to drive improvements, and understand the current adoption of open science practices in a research community. To improve their open science practices, institutions need to measure their performance; however, there is presently no effective system for efficient and large-scale monitoring without significant effort.

Consider the example of open access publishing. A researcher-led large analysis of researchers’ compliance with funder mandates for open access publishing showed that the rate of adherence varied considerably by funder [11]. In Canada, the Canadian Institutes of Health Research (CIHR) had an open access requirement for depositing articles between 2008 and 2015. This deposit requirement was modified when CIHR and the other two major Canadian funding agencies harmonized their policies. The result was a drop in openly available CIHR-funded research from approximately 60% in 2014 to approximately 40% in 2017 [11]. In the absence of monitoring, it is not possible to evaluate the impact of introducing a new policy or to measure how other changes in the scholarly landscape impact open science practices.

The Coronavirus Disease 2019 (COVID-19) pandemic has created increased impetus for, and attention to, open science, which has contributed to the development of new discipline-specific practices for openness [12–14]. The current project aimed to establish a core set of open science practices within biomedicine to implement and monitor at the institutional level (Box 1). Our vision to establish a core set of open science practices stems from the work of Core Outcome Measures in Effectiveness Trials (COMET) [15]. If trialists agree on a few core outcomes to assess across trials, it strengthens the totality of evidence, enables more meaningful use in systematic reviews, promotes meta-research, and may subsequently reduce waste in research. We sought to apply this concept of community-agreed standardization to open science specifically in biomedical research, which currently lacks consensus on best practices, and work to operationalize different open science practices.

## Box 1. Summary of key points

- Funders and other stakeholders in the international research ecosystem are increasingly introducing mandates and guidelines to encourage open science practices.
- Research institutions cannot currently monitor compliance with open science practices without engaging in time-consuming manual processes that many lack the expertise to undertake.
- We conducted an international Delphi study to agree which open science practices would be valuable for research institutions to monitor, with a view to creating an automated dashboard to support monitoring.
- We report 19 open science practices that reached consensus for institutional monitoring in an open science dashboard and describe how we intend to implement these.
- The open science practices identified may be of broader value for developing policy, education, and interventions.

The core set of open science practices identified here will serve the community in many ways, including in developing policy, education, or other interventions to support the implementation of these practices. Most immediately, the practices can inform the development of an automated open science dashboard that can be deployed by biomedical institutions to efficiently monitor adoption of (and provide feedback on) these practices. By establishing what should be reported in an institutional open science dashboard through a consensus building process with relevant stakeholders, we aim to ensure the tool is appropriate to the needs of the community.

## Methodology

### Ethics statement

This study received ethical approval from the Ottawa Health Science Network Research Ethics Board (20210515-01H). Participants were presented with an online consent form prior to viewing round 1 of the Delphi, their completion of the survey was considered implied consent.

For complete study methods, please see S1 Text. We conducted a 3-round modified Delphi survey study. Delphi studies structure communication between participants to establish consensus [16]. Typically, Delphi studies use several rounds of surveys in which participants, experts in the topic area, vote on specific issues. Between rounds, votes are aggregated and anonymized and then presented back to participants along with their own individual scores, and feedback on others’ anonymized voting decisions [17,18]. This gives participants the opportunity to consider the group’s thoughts and to compare and adjust their own assessment in the next round. A strength of this method of communication is that it allows all individuals in a group to communicate their views. Anonymous voting also limits direct confrontation among individuals and the influence of power dynamics and hierarchies on the group’s decision.

Participants in our Delphi were from a convenience sample obtained through snowball sampling of academic institutions interested in open science. The individuals from the institutions represented any/all of the following groups:

1. Library or scholarly communication staff (e.g., responsible for purchasing journal content, responsible for facilitating data sharing or management).
2. Research administrators or leaders (e.g., head of department, CEO, senior management).
3. Staff involved in researcher assessment (e.g., appointment and tenure committee members).
4. Individuals involved in institutional metrics assessment or reporting (e.g., performance management roles).

Because titles and roles differ from institution to institution, we left it to the discretion of the institution to identify participants. Broadly, we aimed to include people who either knew about scholarly metrics or made decisions regarding researcher assessment or hiring. We also explicitly encouraged the institutions to consider diversity of their representing participants (including gender and race) when inviting people to contribute. However, there are a variety of stakeholders that may influence institutional monitoring of open science practices. A limitation of the current work is that we included exclusively participants directly employed by academic institutions. While our intention is to implement the proposed dashboard at biomedical institutions, it is possible we missed nuance or richness, for example, by failing to include representatives from scholarly publishers, academic societies, or funding agencies.

The first two rounds of the Delphi were online surveys administered using Surveylet. Surveylet is a purpose-built platform for developing and administering Delphi surveys [19]. To start with, the Delphi participants were presented with an initial set of 17 potential open science practices to consider that were generated by the project team based on a discussion. Round 3 took the form of two half-day meetings hosted on Zoom [20]. Hosting round 3 in the form of an online meeting is a modification of the traditional Delphi approach. This was done to provide an opportunity for more nuanced discussion among participants about the potential open science practices while still retaining anonymized online voting. We opted for a virtual meeting given the COVID-19 pandemic restrictions at the time and the cost effectiveness for enabling international participation. However, while our use of a modified Delphi in which round 3 took place online provided the opportunity for more nuanced discussion prior to voting, it also meant that we ultimately reduced the overall number of participants taking part in that round in order to host a manageable sized group for the online meeting. This methodological approach may have reduced some of the diversity in potential response despite providing greater richness in responses.

While the structured, anonymous, and democratic approach of the Delphi process offers many advantages to reaching consensus, it is not without limitations. The methods used here may have impacted our outcome. For example, the use of a forced choice item rather than a scale in rounds 2 and 3 may have contributed to a greater likelihood for items to reach consensus in these rounds. While we endeavored to attract a diverse and representative sample of institutions to contribute, ultimately given our sampling approach, it is likely that the participants and institutions that agreed to take part may not be as representative of the global biomedical research culture as we desired, and may have a stronger interest in or commitment to open science than is typical. While the sample may not be generalizable, these institutions likely represent early adopters or willing leaders in open science. Further, our Delphi surveys and consensus meetings were conducted in English only, and the meeting was not conducive for attendance across all time zones. These factors will have created barriers to participation for some institutions or participants. Defining who is an “expert” to provide their views in any Delphi exercise provides an inherent challenge [21]. We faced this challenge here, especially considering the diversity of open science practices and the nuances of applying these practices in distinct biomedical subdisciplines. For example, our vision to create a single biomedical dashboard to deploy at the institutional level may mean we have missed nuances in open science practices in preclinical as compared to clinical research.

### Outcome of the Delphi process

### Round 1

**Participants**: We excluded participants who did not complete 80% or more of the survey in this round. A total of 80 participants from 20 institutions in 13 countries completed round 1. Full demographics are described in Table 1. A total of 44 (55.0%) participants identified as men, 35 (43.8%) as women, and 1 (1.3%) as another gender. Of the 32 research institutions that were invited to contribute to the study, 20 (62.5%) ended up contributing, and 1 to 7 participants from each organization responded to our survey. Researchers (*N =* 31, 38.8%) and research administrators (*N* = 18, 22.5%) comprised most of the sample.

**Table 1:** Characteristics of participants in the Delphi survey.

Participant characteristic	Round 1 N (%)	Round 2 N (%)	Invited for round 3 N (%)	Round 3, day 1 N (%)^a^	Round 3, day 2 N (%)^a^
*Sex*	*N* = 80	*N* = 54	*N* = 50	*N* = 20	*N* = 16
* *Male	44 (55.0)	27 (48.2)	29 (58.0)	9 (45.0)	8 (50.0)
* *Female	35 (43.8)	26 (46.4)	21 (42.0)	11 (55.0)	8 (50.0)
* *Other	1 (1.3)	1 (1.8)	0 (0.00)	0 (0.0)	0 (0.0)
*Age*	*N* = 76	*N* = 51	*N* = 48	*N* = 20	*N* = 16
* *<30	4 (5.3)	1 (1.8)	2 (4.0)	0 (0.0)	0 (0.0)
* *30–40	20 (26.3)	16 (31.4)	13 (26.0)	10 (50.0)	9 (56.3)
* *41–50	20 (26.3)	14 (27.4)	11 (22.0)	5 (25.0)	3 (18.8)
* *51–60	26 (34.2)	18 (35.3)	18 (26.0)	5 (25.0)	3 (18.8)
* *>60	6 (7.9)	2 (3.9)	4 (8.0)	0 (0.0)	0 (0.0)
* *Prefer not to say	0 (0.0)	0 (0.0)	0 (0.0)	0 (0.0)	1 (6.3)
*Racial minority*	*N* = 80	*N* = 54	*N* = 50	*N* = 20	*N* = 16
* *Yes	3 (3.8)	3 (5.4)	0 (0.0)	1 (5.0)	2 (12.5)
* *No	75 (93.8)	49 (87.5)	49 (98.0)	19 (95.0)	14 (87.5)
* *Prefer not to say	2 (2.50)	2 (3.6)	1 (2.0)	0 (0.0)	0 (0.0)
*Research institution*	*N* = 80	*N* = 56	*N* = 50	*N* = 20	*N* = 16
Vall d’Hebron Research Institute (Spain)	6 (7.5)	2 (3.6)	4 (8.0)	0 (0.0)	0 (0.0)
University Vita-Salute San Raffaele Milano (Italy)	7 (8.8)	3 (5.4)	6 (12.0)	0 (0.0)	1 (6.3)
University of Oxford (England)	5 (6.3)	4 (7.1)	3 (6.0)	3 (15.0)	3 (18.8)
University of Nigeria (Nigeria)	4 (5.0)	3 (5.4)	2 (4.0)	0 (0.0)	0 (0.0)
University of Edinburgh (Scotland)	5 (6.3)	4 (7.1)	4 (8.0)	0 (0.0)	0 (0.0)
University of Calgary (Canada)	6 (7.5)	5 (8.9)	4 (8.0)	1 (5.0)	0 (0.0)
University of Basel/University Hospital Basel (Switzerland)	1 (1.3)	2 (3.6)	1 (2.0)	0 (0.0)	0 (0.0)
University Health Network (Canada)	4 (5.0)	3 (5.4)	3 (6.0)	3 (15.0)	2 (12.5)
Universidade Federal de Pelotas (Brazil)	5 (6.3)	4 (7.1)	4 (8.0)	2 (10.0)	3 (18.8)
Universidad de Santiago de Chile (Chile)	5 (6.3)	3 (5.4)	2 (4.0)	0 (0.0)	0 (0.0)
Tanenbaum Open Science Institute (Canada)	5 (6.3)	4 (7.1)	4 (8.0)	2 (10.0)	1 (6.3)
Savitribai Phule Pune University (India)	1 (1.3)	0 (0.0)	1 (2.0)	0 (0.0)	0 (0.0)
Ottawa Hospital Research Institute (Canada)	5 (6.3)	5 (8.9)	3 (6.0)	3 (15.0)	2 (12.5)
Medical University Vienna (Austria)	1 (1.3)	1 (1.8)	1 (2.0)	1 (5.0)	0 (0.0)
King’s Health Partners (England)	2 (2.5)	1 (1.8)	1 (2.0)	0 (0.0)	0 (0.0)
Italian Institute of Technology (Italy)	4 (5.0)	2 (3.6)	3 (6.0)	2 (10.0)	1 (6.3)
Hong Kong Baptist University (Hong Kong)	7 (8.8)	4 (7.1)	3 (6.0)	1 (5.0)	1 (6.3)
Douglas Research Centre (Canada)	4 (5.0)	4 (7.1)	3 (6.0)	1 (5.0)	1 (6.3)
Bond University (Australia)	2 (2.5)	2 (3.6)	0 (0.0)	0 (0.0)	0 (0.0)
University of Turin (Italy)	1 (1.3)	1 (1.8)	1 (2.0)	1 (5.0)	1 (6.3)
*Participant role*	*N* = 80	*N* = 54	*N* = 50	*N* = 20	*N* = 16
Research administrator	18 (22.5)	11 (19.6)	13 (26.0)	5 (25.0)	5 (31.3)
Performance management role (accreditation/bibliometrics/performance/institutional analyst)	4 (5.0)	3 (5.4)	1 (2.0)	2 (10.0)	2 (12.5)
Specialist open science position	4 (5.0)	4 (7.1)	5 (10.0)	4 (20.0)	3 (18.8)
Library or scholarly communication staff	9 (11.3)	4 (7.1)	4 (8.0)	0 (0.0)	0 (0.0)
Researcher (independent researchers, tenured academic staff, faculty involved in research assessment)	31 (38.8)	23 (41.1)	19 (38.0)	8 (40.0)	5 (31.3)
Research support staff (clinical research operations, communications, project manager)	11 (13.8)	8 (14.3)	7 (14.0)	1 (5.0)	1 (6.3)
Trainee (PhD student, graduate trainee)	2 (2.5)	1 (1.8)	1 (2.0)	0 (0.0)	0 (0.0)
Scientific editor	1 (1.3)	0 (0.0)	0 (0.0)	0 (0.0)	0 (0.0)

^a^On each day of the consensus meeting, 1 participant chose not to provide their demographic information.

**Voting**: Of the 17 potential core open science practices presented in round 1, two reached consensus. Participants agreed that “registering clinical trials on a registry prior to recruitment” and “reporting author conflicts of interest in published articles” were essential to include. See full results in Table 2.

**Table 2:** Delphi voting results by round.

		Round 1		Round 2		Round 3	
#	Open science practice	Scale	N (%)^a^	Scale	N (%)^a^	Scale	N (%)^a^
1	Whether clinical trials have been registered before starting recruitment	1–3 4–6 7–9	9 (11.7) 4 (5.2) **64 (83.1)**				
2	Whether systematic reviews have been registered before data collection	1–3 4–6 7–9	6 (7.9) 14 (18.4) 56 (73.7)	Include Exclude Discuss No expertise	36 (75.0) 7 (14.6) 5 (10.4) 10	Include Exclude Abstain	**15 (88.2)** 2 (11.8) 4
3	Whether hypothesis testing research has been registered	1–3 4–6 7–9	17 (22.1) 18 (23.4) 42 (54.5)	Include Exclude Discuss No expertise	26 (51.0) 18 (35.3) 7 (13.7) 5	Include Exclude Abstain	7 (46.7) 8 (53.3) 5
4	Whether any research paper has been registered	1–3 4–6 7–9	13 (16.7) 28 (35.9) 37 (47.4)	Include Exclude Discuss No expertise	15 (28.8) 28 (53.8) 9 (17.3) 4	Include Exclude Abstain	7 (41.2) 10 (58.8) 3
5	Whether data were shared openly at the time of publication (with limited exceptions)	1–3 4–6 7–9	11 (14.1) 13 (16.7) 54 (69.2)	Include Exclude Discuss No expertise	**46 (85.2)** 3 (5.6) 5 (9.3) 3		
6	Whether code was shared openly at the time of publication (with limited exceptions)	1–3 4–6 7–9	4 (5.1) 18 (23.1) 56 (71.8)	Include Exclude Discuss No expertise	**46 (86.8)** 4 (7.5) 3 (5.7) 4		
7	Whether study materials were shared openly at the time of publication (with limited exceptions)	1–3 4–6 7–9	11 (14.1) 17 (21.8) 50 (64.1)	Include Exclude Discuss No expertise	34 (70.8) 6 (12.5) 8 (16.7) 7	Item modified for voting (See #8)	
8^c^	Whether there was a statement about study materials sharing with publications					Include Exclude Abstain	**18 (94.7)** 1 (5.3) 2
9	Reporting the number of preprints	1–3 4–6 7–9	13 (16.9) 21 (27.3) 43 (55.8)	Include Exclude Discuss No expertise	30 (57.7) 13 (25.0) 9 (17.3) 4	Include Exclude Abstain	**15 (88.2)** 2 (11.8) 0
10	Reporting whether articles were published open access at the time of publication	1–3 4–6 7–9	10 (13.0) 14 (18.2) 53 (68.8)	Include Exclude Discuss No expertise	34 (64.2) 8 (15.1) 11 (20.8) 3	Item modified for voting (See #12)	
11	Reporting whether articles were published open access but allowing an embargo period (e.g., 1 year)	1–3 4–6 7–9	23 (30.3) 27 (35.5) 26 (34.2)	Include Exclude Discuss No expertise	28 (53.8) 14 (26.9) 10 (19.2) 4	Item modified for voting (See #12)	
12^c^	Reporting what proportion of articles are published open access with a breakdown of time delay					Include Exclude Abstain	**20 (100)** 0 (0) 1
13	Reporting whether reporting guideline checklists were used	1–3 4–6 7–9	5 (6.4) 13 (16.7) 60 (76.9)	Include Exclude Discuss No expertise	**39 (83.0)** 4 (8.5) 4 (8.5) 10		
14	Reporting whether author conflicts of interests were declared	1–3 4–6 7–9	9 (11.5) 4 (5.1) **65 (83.3)**				
15	Reporting whether author contributions were described	1–3 4–6 7–9	5 (6.4) 17 (21.8) 56 (71.8)	Include Exclude Discuss No expertise	**44 (80.0)** 6 (10.9) 5 (9.1) 1		
16	Reporting on the use of open lab notebooks	1–3 4–6 7–9	18 (23.1) 32 (41.0) 28 (35.9)	Include Exclude Discuss No expertise	13 (28.9) 26 (57.8) 6 (13.3) 12	Include Exclude Abstain	6 (35.3) 11 (64.7) 4
17	Reporting whether ORCID identifiers were used	1–3 4–6 7–9	9 (11.5) 11 (14.1) 58 (74.4)	Include Exclude Discuss No expertise	**48 (87.3)** 5 (9.1) 2 (3.6) 2		
18	Reporting that registered clinical trials were reported in the registry within 2 years of study completion	1–3 4–6 7–9	9 (11.5) 11 (14.1) 58 (74.4)	Include Exclude Discuss No expertise	**45 (93.8)** 1 (2.1) 2 (4.2) 8	Item revoted on with 1 year timeframe (See #19)	
19^c^	Reporting that registered clinical trials were reported in the registry within 1 year of study completion					Include Exclude Abstain	**11 (91.7)** 1 (8.3) 5
20	Reporting the number of replication studies	1–3 4–6 7–9	11 (14.1) 22 (28.2) 45 (57.7)	Include Exclude Discuss No expertise	28 (58.3) 14 (29.2) 6 (12.5) 7	Item modified for voting (See #21 and #22)	
21^c^	Reporting citations to data (refers to data cited in papers)					Include Exclude Abstain	**17 (89.5)** 2 (10.5) 3
22^c^	Reporting citations to code (refers to code cited in papers)					Include Exclude Abstain	14 (77.8) 4 (22.2) 2
23^b^	Reporting on the use of Research Resource Identifiers (RRIDs) (where relevant)			1–3 4–6 7–9	9 (17.3) 25 (48.1) 18 (34.6)	Include Exclude Abstain	7 (53.8) 6 (46.2) 4
24^b^	Reporting whether research articles include funding statements			1–3 4–6 7–9	8 (15.4) 7 (13.5) 37 (71.2)	Include Exclude Abstain	**16 (94.1)** 1 (5.9) 0
25^b^	Reporting whether a published paper has open peer reviews available			1–3 4–6 7–9	9 (17.6) 19 (37.3) 23 (45.1)	Include Exclude Abstain	8 (44.4) 10 (55.6) 2
26^b^	Reporting whether a data management plan was shared			1–3 4–6 7–9	11 (21.6) 15 (29.4) 25 (49.0)	Include Exclude Abstain	4 (21.1) 15 (78.9) 1
27^c^	Reporting whether data/code/materials are shared with a clear license					Include Exclude Abstain	**14 (87.5)** 2 (12.5) 1
28^b^	Reporting whether the data/code/materials license is open or not			1–3 4–6 7–9	6 (12.0) 15 (30.0) 29 (58.0)	Include Exclude Abstain	**13 (81.3)** 3 (18.8) 1
29^b^	Reporting the use of nonproprietary software when sharing data/code/materials			1–3 4–6 7–9	11 (21.6) 19 (37.3) 21 (41.2)	Include Exclude Abstain	6 (54.5) 5 (45.5) 6
30^b^	Reporting the use of persistent identifiers when sharing data/code/materials			1–3 4–6 7–9	5 (9.6) 14 (26.9) 33 (63.5)	Include Exclude Abstain	**15 (88.2)** 2 (11.8) 0
31^b^	Reporting whether workflows in computational environments were shared			1–3 4–6 7–9	8 (15.4) 25 (48.1) 19 (36.5)	Include Exclude Abstain	2 (13.3) **13 (86.7)** 5
32^b^	Reporting the (presumed) gender ratio of the authorship team			1–3 4–6 7–9	23 (44.2) 21 (40.4) 8 (15.4)	Include Exclude Abstain	4 (22.2) 14 (77.8) 2
33^b^	Reporting trial results in a manuscript-style publication (peer reviewed or preprint)			1–3 4–6 7–9	11 (21.2) 19 (36.5) 22 (42.3)	Include Exclude Abstain	**12 (100)** 0 5
34^c^	Reporting systematic review results in a manuscript-style publication (peer reviewed or preprint)					Include Exclude Abstain	**11 (100)** 0 6

^a^Bolded numbers indicate consensus.

^b^Item introduced during round 2.

^c^Item introduced during round 3.

Participants suggested 10 novel potential core open science practices to include in round 2 for voting; they were as follows: use of Research Resource Identifiers (RRIDs) where relevant biological resources are used in a study; inclusion of funder statements; information on whether a published paper has open peer reviews available (definitions vary for open peer review [22], but we define this as having transparent peer reviews available); sharing a data management plan; use of open licenses when sharing data/code/materials; use of nonproprietary software when sharing data/code/materials; use of persistent identifiers when sharing data/code/materials; sharing research workflows in computational environments; reporting on the gender composition of the authorship team; and reporting results of trials in a manuscript-style publication (peer reviewed or preprint) within 2 years of study completion.

### Round 2

**Participants**: Fifty-six (70% of round 1) participants completed the round 2 survey (see Table 1). Of the 20 research institutions that completed round 1, 19 (95%) institutions continued their contributions in round 2, with up to 5 participants from each organization responding to our survey. Researchers (*N =* 23, 41.1%) and research administrators (*N* = 11, 19.6%) again comprised most of the sample, as in round 1.

**Voting**: Of the 15 potential core open science practices that participants had not reached consensus on in round 1, 6 reached consensus in round 2. Participants agreed that the following practices were essential to reporting in the dashboard: whether data were shared openly at the time of publication (with limited exceptions); whether code was shared openly at the time of publication (with limited exceptions); whether reporting guideline checklists were used; whether author contributions were described; whether ORCID identifiers were used; and whether registered clinical trials were reported in the registry within 2 years of study completion.

Participants then ranked the 10 novel potential core open science practices suggested by participants in round 1 for the first time. None of these 10 new practices reached consensus in round 2. There were no other explicitly described practices suggested by participants in round 2 to consider for the dashboard in round 3.

### Round 3

**Participants**: Twenty-one participants were present on day 1 and 17 on day 2 of the consensus meeting. Full demographics are described in Table 1. One participant on each day did not provide any demographic information.

**Voting**: There were 19 items that had not reached consensus in round 2. After discussing each item, some were reworded slightly, expanded into two items, or collapsed into a single item (see notes on modifications made in Table 2). Ultimately, participants voted on 22 potential open science practices in round 3. One of these items asked participants to vote on “reporting whether registered clinical trials were reported in the registry within 1 year of study completion.” An item describing “reporting that registered clinical trials were reported in the registry within 2 years of study completion” reached consensus in round 2; however, several participants commented that the timeframe was inconsistent with requirements of funders that have signed the World Health Organization joint statement on public disclosure of results from clinical trials, which specified 12 months. Based on this, participants were asked to revote on this item using the 1-year cutoff.

Of the 22 potential items voted on in round 3, 12 reached consensus for inclusion: whether systematic reviews have been registered; whether there was a statement about study materials sharing with publications; the use of persistent identifiers when sharing data/code/materials; whether data/code/materials are shared with a clear license; whether the data/code/materials license is open or not; citations to data; what proportion of articles are published open access with a breakdown of time delay; the number of preprints; that registered clinical trials were reported in the registry within 1 year of study completion; trial results in a manuscript-style publication (peer reviewed or preprint); systematic review results in a manuscript-style publication (peer reviewed or preprint); and whether research articles include funding statements. One item reached consensus for exclusion from the dashboard: Reporting whether workflows in computational environments were shared. Participants agreed this item should be a component of the existing item, “reporting whether code was shared openly at the time of publication (with limited exceptions).”

Participants discussed how some of the items that reached consensus for inclusion represented essential practices more broadly related to transparency or reporting than practices generally considered traditional open science procedures. Following round 3, items that reached consensus were grouped based on these broad categories (traditional open science versus broader transparency practices for reporting) and participants were asked to rank the practices based on how they should be prioritized for programming for inclusion in our proposed dashboard (Table 3). Items with higher scores represent those that were given a higher priority. The top two traditional open science practices by priority were reporting whether clinical trials were registered before they started recruitment, and reporting whether study data were shared openly at the time of publication (with limited exceptions). The top two broader transparency practices by priority were reporting whether author contributions were described, and reporting whether author conflicts of interest were described.

**Table 3:** Prioritization of traditional open science practices and broader transparency practices.

No.	Practice	Score
	**Traditional open science practices**	
1	Reporting whether clinical trials were registered before they started recruitment	9.71
2	Reporting whether study data were shared openly at the time of publication (with limited exceptions)	9.18
3	Reporting what proportion of articles are published open access with a breakdown of time delay	8.12
4	Reporting whether study code was shared openly at the time of publication (with limited exceptions)	7.94
5	Reporting whether systematic reviews have been registered before data collection began	6.76
6	Reporting whether clinical trials results appeared in the registry from 1 year after study completion	6.76
7	Reporting whether there was a statement about study materials sharing with publications	6
8	Reporting whether a reporting guideline checklist was used	5.88
9	Reporting citations to data	5.53
10	Reporting trial results in a manuscript-style publication (peer reviewed or preprint)	4.82
11	Reporting the number of preprints	4.35
12	Reporting systematic review results in a manuscript-style publication (peer reviewed or preprint)	2.94
	**Broader transparency practices**	
1	Reporting whether author contributions were described	5.12
2	Reporting whether author conflicts of interest were described	4.71
3	Reporting the use of persistent identifiers when sharing data/code/materials	4.65
4	Reporting whether ORCID identifiers were used	4.47
5	Reporting whether data/code/materials are shared with a clear license	3.47
6	Reporting whether research articles include funding statements	3
7	Reporting whether the data/code/materials license is open or not	2.59

### Consensus core open science practices

Below we briefly consider each of the core practices that reached consensus and discuss the process of implementing each. A total of 19 practices reached consensus for inclusion in the dashboard.

### Traditional open science practices

1. **Reporting whether clinical trials were registered before they started recruitment.** This practice is required by several organizations and funders internationally. Despite clear mandates for registration, we know this practice is not optimal [23]. Standardized reporting of trial registration will allow for linkage of trial outputs to the registry and help contribute to the reduction of selective outcome reporting and non-reporting.
2. **Reporting whether study data were shared openly at the time of publication (with limited exceptions).** Policies encouraging and mandating open data are growing. This practice considers whether there is a statement about open data in a publication. It does not require that this statement indicate that data are in fact publicly available. As culture around data sharing becomes more normative, it may be of value to reevaluate whether tracking the proportion of openly available data is of value. To do so effectively will require changes in the culture around and use of DOIs. Information on the data available and its useability would be essential to provide quality control and for an individual to determine not just if data can be used, but whether it should be used for the intended purpose. Exceptions would include nonempirical pieces (e.g., a study protocol).
3. **Reporting what proportion of articles are published open access with a breakdown of time delay.** This practice reports on the proportion of articles published open access (i.e., publicly available without restriction). Part of this reporting will include the timing of the open access from first publication (e.g., immediate open access versus delayed open access publication).
4. **Reporting whether study code was shared openly at the time of publication (with limited exceptions).** Similar to practice 2, this practice considers whether there is a statement about open code sharing in the publication. It does not require that this statement indicate that code is in fact publicly available. As culture around code sharing becomes more normative, information about the quality and type of code shared and compliance to best practices (e.g., FAIR principles) may be valuable to monitor. Exceptions would include nonempirical pieces.
5. **Reporting whether systematic reviews have been registered.** This practice is required by some journals and is common within knowledge synthesis projects. Standardized reporting of systematic review registration will allow for linkage of review outputs to the registry and help contribute to reduce unnecessary duplication in reviews.
6. **Reporting that registered clinical trials were reported in the registry within 1 year of study completion**. The practice of reporting trial results in the registry they were first registered in is required by several organizations and funders. This practice would track the proportion of trials in compliance with reporting results within 1 year or study completion.
7. **Reporting whether there was a statement about study materials sharing with publications**. This practice considers whether there is a statement about materials sharing with a publication. It does not consider whether or not materials are indeed shared openly. As with data and code sharing, materials sharing is not yet widespread across biomedicine. As a starting point, statements about materials sharing will be monitored, but in time, it may be of value to track the frequency of materials sharing at an institution. This could inform infrastructure needs.
8. **Reporting whether study reporting guideline checklists were used.** Reporting guidelines are checklists of essential information to include in a manuscript; these are widely endorsed by medical journals and have been shown to improve the quality of reporting of publications [24]. This item would track whether reporting guidelines were cited in a publication. In the future, tracking actual compliance to reporting guideline items may be more relevant.
9. **Reporting citations to data.** This practice monitors whether a given dataset shared from researchers at an institution has received citations in other works. This is an assay to data reuse and may be a relevant metric to consider alongside others when considering study impact.
10. **Reporting trial results in a manuscript-style publication (peer reviewed or preprint).** This practice would report whether a trial registered on a trial registry had an associated manuscript-style publication within 1 year of study completion. This will include reporting in the form of preprints.
11. **Reporting the number of preprints.** This practice reports the frequency of preprints produced at the institution over a given timeframe.
12. **Reporting systematic review results in a manuscript-style publication (peer reviewed or preprint).** This practice would report whether a registered systematic review had an associated manuscript-style publication within 1 year of study completion. This will include reporting in the form of preprints.

### Broader transparency practices

1. **Reporting whether author contributions were reported**. Journals are increasingly requiring or permitting authors to make statements (e.g., using the CREDIT Taxonomy) about their role in the publication. This helps to clarify the diversity of contributions each author has made. This practice would track the presence of these statements in publications. Monitoring the use of author contribution statements may help institutions to devise ways to recognize individual’s skills when hiring and promoting researcher.
2. **Reporting whether author conflicts of interest were reported.** Reporting of conflicts of interest is a standard practice at many journals, but this practice is not uniform, with some publications lacking statements altogether. Monitoring conflicts of interest reporting helps to ensure transparency. In the absence of a statement of conflicts of interest, the reader cannot assume none exist. For this reason, we reached consensus that all papers should have such a statement irrespective of whether conflicts exist.
3. **Reporting the use of persistent identifiers when sharing data/code/materials.** Persistent identifiers such as DOIs are digital codes for online objects that remain consistent over time. Use of persistent identifiers of research outputs such as data, code, and materials foster collation and linkage.
4. **Reporting whether ORCID identifiers were reported.** ORCID identifiers are persistent researcher identifiers. This practice would track whether publications report these. Knowledge about use of ORCID will help inform iterations of our open science dashboard. While our dashboard will focus at the research institution level, ORCIDs may be relevant to use to collate institution publications, or to produce researcher-level outputs.
5. **Reporting whether data/code/materials are shared with a clear license.** This practice monitors whether licenses are used when research outputs like data, code, and materials are shared (e.g., use of creative commons licenses).
6. **Reporting whether research articles include funding statements**. Reporting on funding is a standard practice at many journals and required by some funders, but this practice is not uniform, with some publications lacking statements altogether. Monitoring funding statements helps to ensure transparency and provide linkage between funding and research outputs. For this reason, we reached consensus that all papers should have funder statements irrespective of whether funding was received. In the future, knowledge of what types of funding a publication received may foster meta-research on funding allocation and research outputs.
7. **Reporting whether the data/code/materials license is open or not.** Among research outputs shared with a license, this practice monitors the proportion of these that are “open” (i.e., publicly available with no restrictions to access when appropriate to the data).

### Future directions

The next phase of this research program will involve developing the open science dashboard interface and its programming. While we aim to create a fully automated tool, some core open science practices that reached consensus for inclusion in the dashboard may not lend themselves to reliable, automated analysis. For example, the fact that digital identifiers are not widely used on some research outputs (e.g., when sharing code or study materials) may create challenges in accurate measurement. If we find this to be the case, in these instances, we will exclude the open science practice from monitoring. We chose not to restrict the community of Delphi participants in terms of the ease of automation of what they wanted in the tool—we encouraged participants to “think big.” Ultimately, some items may not be possible to include due to feasibility. We anticipate iterative consultation with the community as we work to develop a dashboard that best meets their needs. As infrastructure and the use of identifiers evolve within the biomedical community, there will be a need to refresh consensus and reconsider processes used to best automate the core open science practices.

We anticipate that the open science dashboard will serve as a tool for institutions to track their progress in adopting the agreed open science practices, but also to assess their performance relevant to existing mandates. For example, the dashboard will enable institutions to monitor their adherence to mandates related to open access publishing, clinical trial registration and reporting, and data sharing, all of which are commonly mandated by funders globally and related stakeholders in the research ecosystem [25–27]. We also anticipate that several of the open science practices included in the dashboard will not reflect practices that are widely performed or mandated. Some items may therefore reflect aspirational practices for the community. The dashboard can be used to benchmark for improvements in these areas.

The proposed dashboard is a necessary precursor for providing institutional feedback on the performance of the agreed open science practices. As we pilot implementation of the dashboard, we will consider how the tool can provide tailored feedback to individual institutions, or distinct settings. The central goal of the dashboard is not to facilitate comparison between institutions (i.e., where adherence to practices can be directly compared within the dashboard across different institutions). This type of ranking is counter to our community-driven initiative that seeks to provide a tool for institutional-level improvement in open science rather than to pit organizations, who often are situated quite differently, against one another. Our vision is that the tool will not develop to be punitive, competitive, or a prestige indicator, as this is likely to further contribute to the systematic enablement of high-resource institutions. Nonetheless, a core set of agreed practices is helpful for comparative meta-research around open science.

We intend for the dashboard to be implemented at the individual institution level. Understanding a given institution’s setting, current norms, and resource circumstances will be critical to deciding how to best implement the dashboard in that environment. A key step in the program to develop the proposed dashboard will be to carefully consider the appropriateness of the dashboard being publicly available versus hosted internally by biomedical institutions. Preference is likely to vary across institutions based on their circumstances. As we implement the proposed open science dashboard, it will also be important to measure how nuances in language, geographic location, discipline, and other institutional differences impact optimal local adoption. Even subtle differences in understanding of, and experiences with, open science at different institutions may have an important impact on how an eventual dashboard can be implemented to best meet institutional needs while still retaining a core set of practices to monitor.

Over time, we will also need to monitor the dashboard itself. As open science becomes increasingly embedded in the research ecosystem, the core practices of today may differ from those of the future. During implementation, we will evaluate how the tool is impacted by subtleties and practical constraints differing between institutions, countries, and geographical regions (for example, how appropriate the tool is in a Global North versus Global South setting). Addressing the distinct challenges will help to foster harmonization in measuring open science practices in the biomedical community. We will need to monitor and stay abreast of the global communities needs and practices to ensure the dashboard is sustainable and relevant over time.

## Conclusions

This Consensus View aimed to establish a core set of open science practices to monitor at biomedical institutions. The core set of practices was developed to inform items to track in a proposed automated open science dashboard, which could be deployed by institutions and report aggregated institutional-level information on performance for each practice included. The value of a consensus list of open science practices may be of broad value when developing policy, education, and interventions to improve open science in biomedicine.

Through consulting with 80 stakeholders from 20 institutions, consensus was reached on the value of tracking 19 practices in the proposed dashboard. By taking the approach of consulting the community and building consensus on the practices to include in the dashboard, we intend to develop a dashboard that best meets the needs of the community. By bringing the community together prior to developing the tool, we have also had the opportunity to brainstorm and discuss implementation strategies. We now have a roadmap to guide how to obtain community feedback on a prototype of the dashboard and a plan to pilot implementation at 3 institutions. This pilot and implementation exercise will position us to better understand barriers and enablers to adoption and use of the proposed open science dashboard [28].

## Supporting information

S1 Text Full Delphi protocol. (DOCX)

## Abbreviations

CIHR: Canadian Institutes of Health Research
COMET: Core Outcome Measures in Effectiveness Trials
COVID-19: Coronavirus Disease 2019
RRID: Research Resource Identifier
