# Supplementary material for: Community consensus on core open science practices to monitor in biomedicine
Source: PLoS Biol. 2023 Jan 24;21(1):e3001949. doi: 10.1371/journal.pbio.3001949 (PMC9873153; doi:10.1371/journal.pbio.3001949)
Supplement: S1 Text — (DOCX) [file pbio.3001949.s001.docx]

**Supplement 1.** Full Delphi protocol

**Ethics Statement**

This study received ethical approval from the Ottawa Health Science Network Research Ethics Board (20210515-01H). Participants were presented with an online consent form prior to viewing round 1 of the Delphi, their completion of the survey was considered implied consent.

**Transparency Statement**

The study protocol was posted on the Open Science Framework^28^ prior to data collection and has drawn some of the text of this Methods section directly from the protocol. All related study materials and data are available at: 10.17605/OSF.IO/JM8WG^29^.

**Study Design**

We conducted a 3-round modified Delphi survey study. Delphi studies structure communication between participants to establish consensus^16^. Typically, Delphi studies use several rounds of surveys in which participants, experts in the topic area, vote on specific issues. Between rounds, votes are aggregated and anonymized and then presented back to participants along with their own individual scores, and feedback on others’ anonymized voting decisions^17,18^. This gives participants the opportunity to consider the group’s thoughts and to compare and adjust their own assessment in the next round. A strength of this method of communication is that it allows all individuals in a group to communicate their views. Anonymous voting also limits direct confrontation among individuals and the influence of power dynamics and hierarchies on the group’s decision.

The first two rounds of the Delphi were online surveys administered using Surveylet. Surveylet is a purpose-built platform for developing and administering Delphi surveys^19^. Round 3 took the form of two half-day meetings hosted on Zoom. Hosting Round 3 in the form of an online meeting is a modification of the traditional Delphi approach. This was done to provide an opportunity for more nuanced discussion among participants about the potential open science practices while still retaining anonymized online voting. We opted for a virtual meeting given the COVID-19 pandemic restrictions at the time and the cost effectiveness for enabling international participation.

**Recruitment of Participants**

The project team generated an initial list of 32 research institutions from discussion based on our networks who we felt were directly or indirectly interested in monitoring open science practices in biomedicine. We then snowball sampled (i.e., an iterative process of selecting institutions; the process is often started with a small number of institutions that meet specific inclusion criteria) from this list by asking leaders at interested institutions to connect us with their peers at other institutions who may be interested in participating. We explicitly asked institutional leaders we contacted to consider geographic representation in their suggestions. This approach was used to ensure that the institutions we involved has some existing interest in open science and leadership that would be open to pilot testing the dashboard tool.

The snowball sampling approach generated a list of 32 institutions from 22 countries and institutional contacts in leadership, or contacts who could communicate our invitation to leadership (see table below). We contacted each institution to describe our study and invite them to participate. If an institution confirmed interest in participating, we asked them to identify 4–6 members of their research community from any/ all of the following groups to take part in the Delphi:

1. Library or scholarly communication staff (e.g., responsible for purchasing journal content, responsible for facilitating data sharing or management)
2. Research administrators or leaders (e.g., head of department, CEO, senior management)
3. Staff involved in researcher assessment (e.g., appointment and tenure committee members)
4. Individuals involved in institutional metrics assessment or reporting (e.g., performance management roles)

Because titles and roles differ from institution to institution, we left it to the discretion of the institution to identify participants. Broadly, we aimed to include people who either knew about scholarly metrics, or who made decisions regarding researcher assessment or hiring. We also explicitly encouraged the institutions to consider diversity of their representing participants (including gender, race) when inviting people to contribute. Institutions were sent reminders to respond after 1 and 2 weeks, respectively to confirm their interest and to have their representative participants complete the initial survey. To acknowledge participants’ time for taking part in the study, they had the opportunity to enter their name into a draw to win an iPad.

*Round 1*

Participants were asked to complete an 8-item survey via Surveylet (see below). This survey was pilot tested for useability, and we estimated a survey completion time of 15 min. The survey gathered participant demographics, information about their institution and their role within it. Then, the survey presented participants with a list of 17 open science practices potentially relevant to include in an automated dashboard for biomedical research institutions. This list was developed by the project team. Each open science practice was defined to ensure clarity and that participants had standardized information. Participants were asked to indicate the extent to which they agreed with the inclusion of each practice in a list of core open science practices tracked in the proposed institutional dashboard. We stressed that we sought to include only practices of broad applicability and high priority in the implementation of open science in biomedicine. We instructed participants not to consider the technical feasibility of automating the presented open science practices for the proposed dashboard. Participants responded on a 9-point Likert scale with endpoints labeled ‘strongly disagree’ (1) to ‘strongly agree’ (9). Participants were also provided with a textbox after each item to provide any additional information or to justify their answer. The last part of the survey invited participants to describe additional open science practices which they believed should be considered core that were not already presented and to offer any additional feedback on the survey.

Scores for each of the survey items on Round 1 were aggregated and analyzed. All items in which 80% of respondents felt the practice should be included or excluded were removed from voting in round 2. We defined consensus as 80% of responses being in the top third (7-9) or bottom third (1-3) of the 9-point scale. We chose 80% based on findings from a systematic review of Delphi studies^30^.

*Round 2*

For the complete Round 2 survey please see below. All participants who completed Round 1 of the Delphi were re-invited to participate in Round 2. Any items that did not reach consensus in Round 1 were presented again in Round 2. Participants were first presented with the practice under consideration, the aggregate group score for that practice from Round 1, their own score for that practice from Round 1, and comments provided by participants. They were then asked to indicate if the practice should be: (i) ‘included in the dashboard’; (ii) ‘excluded from the dashboard’; (iii) ‘discussed at the consensus meeting’, or to indicate that they (iv) ‘don’t have expertise related to the topic’. Participants were also provided with all new open science practices suggested by participants in Round 1. In total 10 new practices were presented in Round 2. Each potential new item was briefly defined to ensure clarity and standardized information about the practice. As in Round 1, it was stressed that the dashboard sought to include only open science practices that were essential to implementing open science in biomedicine. Responses to the new items were provided on the same 9-point Likert scale used in survey 1, with endpoints labeled ‘strongly disagree’ (1) to ‘strongly agree’ (9). Again, participants could provide additional information or justify their answer. At the end of the survey, participants could again suggest new items to include, and provide any additional feedback on the survey.

*Round 3*

For the list of items voted on in round 3, please see below. We randomly selected 50 of the 80 respondents from Round 1 to invite to participate in Round 3 (using the RAND function in Excel). Given that Round 3 required virtual attendance at two half-day meetings, we anticipated approximately half of those invited would participate which would provide a feasible group size for online discussion. We made slight modifications to our list of randomly selected invitees to ensure all institutions were sufficiently represented. Specifically, we added a randomly selected participant from any institution not represented in the list and when doing so removed a random participant from the institution with highest representation. Due to time-zone scheduling difficulties we replaced all participants from Australia. The materials used for the meeting, including the agenda, have been shared on the Open Science Framework.

To minimize the potential for bias in the discussion introduced by the core research team, the online meeting was moderated by a researcher (CLA) experienced with Delphi studies and familiar with research assessment, but who was not part of the initial core research team. On day 1 we presented the results of Rounds 1 and 2 of the Delphi survey, and highlighted items that reached consensus. We also presented a prototype of what the dashboard tool could look like (https://quest-dashboard.charite.de/). Then, we gave participants the opportunity to engage in a general discussion about the project. Following this, the items not yet in consensus were grouped according to themes and presented for discussion and voting for inclusion/exclusion in the dashboard. Participants voted on each of the items using Zoom polling software. Responses were recorded using the following options: (i) “include”, (ii) “exclude”, or (iii) “abstain”. In accordance with Rounds 1 and 2, consensus was prospectively defined as 80% of the responses in the include or exclude category, after removal of “abstain” responses. The wording of some items was modified based on discussion to better reflect nuances in the views of the group prior to voting.

After voting was completed on all potential open science practices, we facilitated a focused discussion on how to best implement the proposed open science dashboard. An expert (JB) in implementing complex interventions based on French’s Framework^23^ for theory-informed behaviour change interventions provided a presentation to offer context. This was followed by a brainstorming exercise structured around addressing the four steps of French’s Framework in regards to implementing the open science dashboard: Step 1) Who needs to do what, differently? ; Step 2) Which barriers and enablers need to be addressed?; Step 3) Which intervention components could overcome the modifiable barriers and enhance the enablers?; Step 4) How can behaviour changes be measured and understood? Following the second day of the meeting, participants were sent the list of the core open science practices established through the Delphi process and asked to rank order the relative importance of each for prioritizing in the proposed dashboard. Based on discussions on day 2 of the meeting, items were split into two categories, ‘traditional open science practices’ and broader ‘transparency practices’.

**Protocol Amendments**

We had indicated that we would post information about our study, to recruit new institutions, to the Declaration on Research Assessment (DORA) newsletter. This was not pursued as we felt our snowball sampling from our originally generated list was sufficient. Sticking to our snowballing approach also allowed us to retain a measure of the population size of institutions invited and the response rate.

**Table 1.** List of institutions initially identified and invited to contribute

| **No.** | **Institution** | **Country** |
| --- | --- | --- |
| 1 | Bond University | Australia |
| 2 | Queensland University of Technology | Australia |
| 3 | Medical University Vienna | Austria |
| 4 | UZ Leuven | Belgium |
| 5 | Universidade Federal de Pelotas | Brazil |
| 6 | Ottawa Hospital Research Institute | Canada |
| 7 | University of Calgary | Canada |
| 8 | University Health Network | Canada |
| 9 | Montreal Neurological Institute | Canada |
| 10 | Douglas Research Centre | Canada |
| 11 | University of Santiago | Chile |
| 12 | Universidad del Rosario | Colombia |
| 13 | University of Oxford | England |
| 14 | King’s Health Partners | England |
| 15 | UPMC-Paris 06 University | France |
| 16 | Charité Universitätsmedizin Berlin | Germany |
| 17 | University of Hong Kong | Hong Kong, China |
| 18 | Hong Kong Baptist University | Hong Kong, China |
| 19 | Pune University | India |
| 20 | Università Vita-Salute San Raffaele | Italy |
| 21 | University of Torino | Italy |
| 22 | Istituto Italiano di Tecnologia | Italy |
| 23 | National Autonomous University of Mexico | Mexico |
| 24 | Erasmus University Medical Center Amsterdam | Netherlands |
| 25 | University of Nigeria | Nigeria |
| 26 | University of Edinburgh | Scotland |
| 27 | Stellenbosch University | South Africa |
| 28 | Vall d’Hebron Institut de Recerca | Spain |
| 29 | Karolinska University Hospital | Sweden |
| 30 | University of Basel / University Hospital Basel | Switzerland |
| 31 | Stanford University | United States of America |
| 32 | University of Alabama | United States of America |

**Table 2. Round 1 survey**

1. What is your age?
2. Which describes you best?
   Choices: female, male, transgender, non-binary, other, prefer not to say
3. Do you identify as being part of an ethnic or racial minority group?
   Choices: yes, no, prefer not to say
4. Which organization is your primary affiliation?
5. Which describes you best?
   Choices: early-career researchers (within 5 years of first permanent position); mid-career researchers (5-10 years into permanent position); senior researchers (more than 10 years into their permanent position); library or scholarly communication staff; research administrators; faculty involved in researcher assessment (e.g., hiring and tenure committee members); other, please specify

*The aim of this project is to come up with a core set of open science practices that biomedical institutions can track over time. Once we agree on what core open science practices are important in the community, we plan to develop an automated online dashboard to track these practices over time. By tracking open science practices, we will be better able to create open science policies and measure the impact of these policies and other interventions to drive improvements in open science practices.*

*There are a wide range of possible open science practices. For each of the practices below, please indicate how important you think each is to be included in our core set of open science practices. We define core open science practices as those that are essential to perform for virtually all studies.*

1. Please indicate your response on the 1-9 scale provided (1 = Strongly disagree that this is a core open science practice; 5 =Unsure if this is a core open science practice; 9 = Strongly agree that this is a core open science practice). Please provide any comments about the item or your decision that you would like to share with the other participants.

| Practice | Description |
| --- | --- |
| Registering protocols for clinical trials | Study registration is when the methods of a proposed study (e.g., protocol) are posted publicly prior to starting the study or the study analysis. There are several existing mandates to register clinical trial studies in clinical trial databases. This is done to enhance transparency and reduce duplication, publication bias, and selective outcome reporting. See examples [here](https://www.who.int/clinical-trials-registry-platform) and [here](https://clinicaltrials.gov/). |
| Registering protocols for systematic reviews | Study registration is when the methods of a proposed study (e.g., protocol) are posted publicly prior to starting the study or the study analysis. There are strong norms to register systematic reviews and other knowledge synthesis projects in databases. This is done to reduce duplication, publication bias, and selective outcome reporting. See for example [here](https://www.crd.york.ac.uk/PROSPERO/) and [here](http://syrf.org.uk/protocols/). |
| Registering protocols for all hypothesis testing research studies | Study registration is when the methods of a proposed study (e.g., protocol) are posted publicly prior to starting the study or the study analysis. Increasingly, researchers are publishing study protocols for projects beyond those using a clinical trial or systematic review design. This is done to enhance transparency and to reduce duplication, publication bias, and selective outcome reporting. This can be done using tool such as the [Open Science Framework.](https://www.cos.io/products/osf) In psychology, this process is often referred to as “[pre-registration](https://www.cos.io/initiatives/prereg)”. The argument has been made that all research that tests a hypothesis should be registered to help reduce bias and selective reporting. |
| Registering protocols for all research studies | Study registration is when the methods of a proposed study (e.g., protocol) are posted publicly prior to starting the study or the study analysis. Increasingly, researchers are publishing study protocols for projects beyond those using a clinical trial or systematic review design. This can be done using tool such as the [Open Science Framework.](https://www.cos.io/products/osf) In psychology, this process is often referred to as “[pre-registration](https://www.cos.io/initiatives/prereg)”. The argument has been made that all studies, even exploratory or discovery research, should be registered to help enhance transparency and to reduce duplication, publication bias, and selective outcome reporting. |
| Sharing study data openly at the time of publishing with limited exceptions | [CASRAI](https://dictionary.casrai.org/Data) defines data as "Facts, measurements, recordings, records, or observations about the world collected by scientists and others, with a minimum of contextual interpretation. Data may be in any format or medium taking the form of writings, notes, numbers, symbols, text, images, films, video, sound recordings, pictorial reproductions, drawings, designs or other graphical representations, procedural manuals, forms, diagrams, work flow charts, equipment descriptions, data files, data processing algorithms, or statistical records." Several jurisdictions have created policies encouraging data sharing. See examples [here](https://www.nihr.ac.uk/documents/nihr-position-on-the-sharing-of-research-data/12253) and [here](https://grants.nih.gov/grants/policy/data_sharing/data_sharing_guidance.htm). Data sharing involves making research data openly available for others to access and build upon freely.  Data sharing is thought to promote transparency, support reproducibility, and foster innovation. In biomedicine, there are limited exceptions in which full open data sharing is not acceptable (e.g., studying a rare disease where anonymous data could still be identifiable; archival data where patient consent for sharing was not obtained). |
| Sharing study code at the time of publishing | Sharing code refers to the practice of sharing the information from computer programs that allowed us to perform our research. This may include scripts that help analyze our data. Sharing code, especially when it comes from open source or widely available programs, can allow programs to be scrutinized and results to be replicated. Sharing of study code is often seen as part of the responsibilities associated with data sharing. |
| Sharing study materials at the time of publishing | Sharing study materials refers to the practice of physical research materials needed to conduct a study. In biomedicine there are a range of study materials that may be relevant to share including biological materials like cell lines and antibodies. Many disciplines have established repositories that support the sharing of study materials, see for example the [Knockout Mouse Project](https://www.komp.org/redirect.html) or the [Cancer Cell Line Encyclopedia.](https://portals.broadinstitute.org/ccle) |
| Creating preprints | Preprints are versions of manuscripts that are shared publicly, typically prior to submission to a journal for publication. They are not peer reviewed. Preprints can be created for free using a number of different preprint servers including [MedRxiv](https://www.medrxiv.org/). |
| Publishing research in an open access format immediately | Open access publishing refers to the process of sharing work in a way that is free to access and uses copyright licensing that creates no or limited barriers to build on the work. Several jurisdictions have mandates for open access publishing, see [here](https://ec.europa.eu/info/research-and-innovation/strategy/goals-research-and-innovation-policy/open-science/open-access_en#:~:text=In%202012%2C%20the%20European%20Commission,and%20the%20knowledge%2Dbased%20economy.&text=reporting%20on%20the%20follow%2Dup.) and [here](https://www.nih.gov/health-information/nih-clinical-research-trials-you/what-is-nih-public-access-policy#:~:text=The%20Public%20Access%20Policy%20ensures,.gov%2Fpmc%2F).), for examples. [Plan S](https://www.nih.gov/health-information/nih-clinical-research-trials-you/what-is-nih-public-access-policy#:~:text=The%20Public%20Access%20Policy%20ensures,.gov%2Fpmc%2F).), a coalition of national funding organizations, has recently called for open access publishing to occur for all publicly funded research without delay. Some jurisdictions, including [Canada](https://cihr-irsc.gc.ca/e/32005.html), permit a 12 month embargo period prior to the requirement of open access. |
| Publishing research in an open access format but allowing an embargo period (E.g., within 12 months of publication the work must be open access) | Open access publishing refers to the process of sharing work in a way that is free to access and uses copyright licensing that creates no or limited barriers to build on the work. Several jurisdictions have mandates for open access publishing, see [here](https://ec.europa.eu/info/research-and-innovation/strategy/goals-research-and-innovation-policy/open-science/open-access_en#:~:text=In%202012%2C%20the%20European%20Commission,and%20the%20knowledge%2Dbased%20economy.&text=reporting%20on%20the%20follow%2Dup.) and [here](https://www.nih.gov/health-information/nih-clinical-research-trials-you/what-is-nih-public-access-policy#:~:text=The%20Public%20Access%20Policy%20ensures,.gov%2Fpmc%2F).), for examples. [Plan S](https://www.nih.gov/health-information/nih-clinical-research-trials-you/what-is-nih-public-access-policy#:~:text=The%20Public%20Access%20Policy%20ensures,.gov%2Fpmc%2F).), a coalition of national funding organizations, has recently called for open access publishing to occur for all publicly funded research without delay. Some jurisdictions, including [Canada](https://cihr-irsc.gc.ca/e/32005.html), permit a 12-month embargo period prior to the requirement of open access. |
| Use of reporting guideline checklists | Reporting guidelines are checklists of essential information that ought to be reported in a manuscript. There are different reporting guidelines for different biomedical research designs. See for example the [CONSORT](http://www.consort-statement.org/) guideline for randomized trials, or the [ARRIVE](https://arriveguidelines.org/) guideline for reporting preclinical research studies. Reporting guidelines are widely endorsed by hundreds of biomedical journals and there is evidence that their use effectively increases reporting quality. |
| Use of open lab notebooks | Globally an increasing number of researchers are making their research available on a day-to-day basis through the curation of an open digital lab book, see for example [here](https://openlabnotebooks.org/). This practice is thought to increase transparency, reduce duplication, and foster collaboration. |
| Engaging in replication studies | Interest in, and recognition of, the value of direct replications of existing studies has received growing attention. Little is known about the proportion of studies that are replicated within biomedicine or what replication outcomes are. |
| Reporting author conflicts of interest when publishing | Conflict of interest are situations where an individual’s involvement with another individual or organization, whether personal, financial, or otherwise, may have an impact on their bias or the perception of bias on a topic. Reporting conflicts of interest is a mandatory practice at many biomedical journals, but compliance rates are unknown. Reporting conflicts of interest help to increase transparency. |
| Reporting author contributions when publishing | Increasingly journals are allowing authors the option to specify how each named author contributed to the research project by describing their role. Tools such as [the Credit taxonomy](https://casrai.org/credit/) have helped foster transparency and provide a more nuanced look at author credit. |
| Use of ORCID identifiers on publications | [ORCID identifiers](https://orcid.org/) are persistent digital identifiers for researchers that help openly distinguish between individuals and track their research outputs. |
| Reporting results of trails in the trial registry within 2-years of study completion | Several jurisdictions require that clinical trial results be populated in the registry where they were initially registered, often within a 2-year timeframe. For examples, please see [here](https://www.who.int/clinical-trials-registry-platform) and [here](https://clinicaltrials.gov/).  This is to create a public record of the results but also the create a comprehensive record of the study from start to finish and in a standardized reporting format. |

Note: for each item above participants will be presented with a comment box in which they can choose to explain their reasoning for their vote.

1. Are there any open science practices that you think are core to monitor that were not listed in the previous section? If so, please describe these here.
2. Do you have any other comments to share? If so, please describe these here.

**Round 2 Survey**

**Section 1.**

For items in this section, you are asked to use the scale:

- Exclude this open science practice from monitoring
- Include this open science practice for monitoring
- I want to discuss this practice further in the virtual meeting as part of the 3^rd^ round of the Delphi
- I don’t have enough content expertise to vote on this item

For each item you have the option of providing a comment to justify your answer.

**Note that the table of definitions provided in the Round 1 survey was again provided.**

1. Registering protocols for systematic reviews
2. Registering protocols for all hypothesis testing research studies
3. Registering protocols for all research studies
4. Sharing study data openly at the time of publishing with limited exceptions
5. Sharing study code at the time of publishing
6. Sharing study materials at the time of publishing
7. Creating preprints
8. Publishing research in an open access format immediately (ie. Gold Open Access)
9. Publishing research in an open access format but allowing an embargo period (i.e., Green Open Access)
10. Use of reporting guideline checklists
11. Use of open lab notebooks
12. Engaging in replication studies
13. Reporting author contributions when publishing
14. Use of ORCID identifiers on publications
15. Reporting results of trials in the trial registry within 2-years of study completion

**Section 2.**

For items in this section, you are asked to respond indicating how much you agree the item should be included in the open science dashboard, on a 1 to 9 scale, with endpoints and midpoint:

1. Strongly Disagree 5- Unsure 9- Strongly agree

For each item you have the option of providing a comment to justify your answer.

1. Use of #RRID (Research Resource Identifiers) where relevant biological resources are used in a study.

*Research Resource Identifiers (#RRID) are ID numbers assigned to help researchers cite key resources (antibodies, model organisms and software projects) in the biomedical literature to improve transparency of research methods.*

1. Inclusion of funder statements.

*Funder statements ought to specify whether the research was funded or not, if so, who the funder is and what role they had in the design and conduct of the work.*

1. Information of whether a published paper has open peer reviews available.

*Open peer review refers to the process of making peer reviewer, editorial, and author rebuttals feedback publicly available alongside a published article. Depending on the journal sometimes the names of the peer reviewer and editor are published alongside their review reports. One value of open peer review is increased transparency regarding journal operations and decision making.*

1. Sharing a data management plan.

*Data management plans are documents that describe how a given research project will acquire, manage, analyze, store, share and archive data resulting from the work. Some stakeholders, such as federal funders in some jurisdictions, require data management plans.*

1. Use of open licenses when sharing data/code/materials.

*Sharing data/code/materials using a CC-0, CC-BY, or similar license reduces barriers for others to access, use, and built upon shared materials. Clear use of a creative Commons license means that others may not have to contact the authors of the data/code/materials to enquire about permissions for reuse or modification.*

1. Use on non-proprietary software when sharing data/code/materials.

*Non-proprietary software refers to software that are in the public domain that do not require licenses to access and use. Sharing data/code/materials using non-proprietary software may foster greater access.*

1. Use of persistent identifiers when sharing data/code/materials.

*Persistent identifiers are permanent references to a document. An example of a persistent identifier is a DOI. Sharing data/code/materials using persistent identifiers helps make them findable, recognizable, and more easily mapped between information systems.*

1. Sharing of research workflows in computational environments.

*For biomedical research (e.g., bioinformatics) involving complex analytical pipelines, the use of workflow tools can be used to transparently present processes. E.g., Snakemake, Nextflow, Galaxy, and Apache Taverna.*

1. Gender of authorship team.

*Many institutions and jurisdictions are increasingly focused on equity, diversity, and inclusion in research. One aspect of this involves supporting female researchers to continue in research and in research leadership. This metric would report the overall ‘female’ contribution to the research project, e.g., the proportion of team members based on author name, that are likely to be female.*

1. Reporting trial results in a manuscript within 2 years of study completion

*Reporting result of trials in a manuscript-style publication (peer-reviewed or preprint) within 2-years of study completion.*

**Round 3 Survey**

1. What is your age?
2. Which describes you best? (female, male, transgender, non-binary, other, prefer not to say)
3. Do you identify as being part of an ethnic or racial minority group (yes, no, prefer not to say)
4. Which organization is your primary affiliation?
5. Which describes you best? (early-career researchers (within 5 years of first permanent position); mid-career researchers (5-10 years into permanent position); senior researchers (more than 10 years into their permanent position); library or scholarly communication staff; research administrators; faculty involved in researcher assessment (e.g., hiring and tenure committee members); other, please specify.)

*Day 1*

Items were voted on using the responses: include, exclude and abstain.

1. A metric reporting what proportion of articles are published open access with a breakdown of time delay
2. A metric reporting whether systematic reviews have been registered
3. A metric reporting whether hypothesis testing research has been registered
4. A metric reporting whether any research paper has been registered
5. A metric reporting whether there was a statement about study materials sharing with publications
6. A metric reporting whether open lab notebook information has been shared
7. A metric reporting citations to data
8. A metric reporting citations to code
9. A metric reporting whether a published paper has open peer reviews available
10. A metric reporting whether a data management plan has been shared in a published article
11. A metric reporting whether workflows in computational environments were shared
12. A metric reporting the (presumed) gender ratio of the authorship team

*Day 2*

Items were voted on using the responses: include, exclude and abstain.

1. A metric reporting the number of preprints
2. A metric reporting whether research articles include funding statements
3. A metric reporting the use of persistent identifiers when sharing data/code/materials
4. A metric reporting the use of Research Resource Identifiers (RRID) (where relevant)
5. A metric reporting whether data/code/materials are shared with a clear license
6. A metric reporting whether the data/code/materials license is open or not
7. A metric reporting the use of non-proprietary software when sharing data/code/materials
8. A metric reporting that registered clinical trials were reported in the registry within 1 year of study completion
9. A metric reporting trial results in a manuscript-style publication (peer reviewed or preprint)
10. A metric reporting systematic review results in a manuscript-style publication (peer reviewed or preprint)

**Post consensus meeting ranking survey**

Below you will find two questions. The first presents what we considered to be traditional open science practice, while the second presents indicators that may be more broadly related to transparency. Please rank order the items in each list based on which you think is most relevant to prioritize for inclusion in the dashboard.

- 1. Traditional Open Science Practices: Please rank order the following items that reached consensus based on which you think is of most importance to include in the dashboard. To do so you can click and drag the items up or down.

| A metric reporting whether clinical trials were registered before they started recruitment |
| --- |
| A metric reporting whether study data was shared openly at the time of publication (with limited exceptions) |
| A metric reporting whether study code was shared openly at the time of publication (with limited exceptions) |
| A metric reporting whether study reporting guideline checklists were used |
| A metric reporting whether systematic reviews have been registered |
| A metric reporting whether there was a statement about study materials sharing with publications |
| A metric reporting citations to data |
| A metric reporting what proportion of articles are published open access with a breakdown of time delay |
| A metric reporting the number of preprints |
| A metric reporting that registered clinical trials were reported in the registry within 1 year of study completion |
| A metric reporting trial results in a manuscript-style publication (peer reviewed or preprint) |
| A metric reporting systematic review results in a manuscript-style publication (peer reviewed or preprint) |

- 1. Open science practices related to reporting transparency: Please rank order the following items that reached consensus based on which you think is of most importance to include in the dashboard. To do so you can click and drag the items up or down.2

| A metric reporting whether author conflicts of interest were reported |
| --- |
| A metric reporting whether author contributions were reported |
| A metric reporting whether ORCID identifiers were reported |
| A metric reporting the use of persistent identifiers when sharing data/code/materials |
| A metric reporting whether research articles include funding statements |
| A metric reporting whether data/code/materials are shared with a clear license |
| A metric reporting whether the data/code/materials license is open or not |
